# An Assessment of Snail-Farm Systems Based on Land Use and Farm Components

**DOI:** 10.3390/ani11020272

**Published:** 2021-01-21

**Authors:** Konstantinos Apostolou, Alexandra Staikou, Smaragda Sotiraki, Marianthi Hatziioannou

**Affiliations:** 1 Department of Ichthyology & Aquatic Environment, Faculty of Agricultural Sciences, University of Thessaly, Fytoko Street, 38 445 Nea Ionia Magnesia, Greece; 2 Department of Zoology, School of Biology, Aristotle University of Thessaloniki, 54124 Thessaloniki, Greece; astaikou@bio.auth.gr; 3 Veterinary Research Institute, Hellenic Agricultural Organization DEMETER, HAO Campus, 57001 Thermi, Greece; sotiraki@vri.gr

**Keywords:** heliciculture, invertebrate livestock, environmental sustainability, typology

## Abstract

**Simple Summary:**

This study’s goal was a thorough analysis and a detailed characterization of commercial snail farms in Greece, considering the unstructured development of the snail-farming sector over recent years. Additionally, the characterization of snail farms in Greece could help Southern European countries improve heliciculture. This study classifies 29 farms in five snail farming systems: elevated sections (7%), net-covered greenhouse (38%), a mixed system with a net-covered greenhouse (10%), open field (38%), and mixed system with an open field (7%). Results showed the impact of various parameters (farming system, region, equipment, and facilities) on annual production. Snail farms were dispersed in six different regions (Thrace, Central Macedonia, West Macedonia, Thessaly, Western Greece, and the Attica Islands). The location affected productivity, but also influenced the duration of operation during an annual cycle.

**Abstract:**

In this study, the structural and management characteristics of snail farms in Greece were analyzed to maximize sustainable food production. Objectives, such as the classification of farming systems and assessing the effects of various annual production parameters, were investigated. Data were collected (2017) via a questionnaire, and sampling was conducted in 29 snail farms dispersed in six different regions (Thrace, Central Macedonia, West Macedonia, Thessaly, Western Greece, and the Attica Islands). Descriptive statistics for continuous variables and frequencies for categorical variables were calculated. The similarity between farms was analyzed using nonmetric multidimensional scaling (nMDS). The average farm operation duration exceeded eight months and the mean annual production was 1597 kg of fresh, live snails. Results recorded five farming systems: elevated sections (7%), net-covered greenhouse (38%), a mixed system with a net-covered greenhouse (10%), open field (38%), and mixed system with an open field (7%). Snail farms differ in the type of substrate, available facilities, and equipment (60% similarity between most of the open field farms). The geographical location of a farms’ settlement affects productivity but also influences the duration of operation, especially in open field farms, due to their operation under a wide assortment of climatic types.

## 1. Introduction

Heliciculture has been practiced since the 1st century BC. Nowadays, commercial snail farms have been established in many parts of the world. Simultaneously, extensive and intensive farming methods have been developed [1,2,3]. Terrestrial gastropods are a high-value food product and a source of special byproducts (caviar, mucus, and bioactive substances) with great commercial value [4,5]. In recent years, in the dermatological sector, there has been an increase in the use of snail extract (e.g., in management of burns) as it has exhibited therapeutic, sedative, and anti-aging properties [4,5]. Universally, consumable invertebrates, including snails, could be regarded as a significant protein source for a globally increasing population, which is expected to reach nine billion people in 2050 [6].

European consumption of edible snails exceeds 100,000 tons, with overall imports displaying a 49% increase between 1995 and 2010 [7,8]. As a development activity in Greece, heliciculture constitutes an alternative source of income even in areas with farmland of low productivity [9,10]. According to the latest available data from the Hellenic Ministry of Rural Development and Food [11] in Greece, 131 snail farms are in operation, occupying 578,000 m^2^. Of these, 75 (57%) are the open-air type, which occupies an area of 482,000 m^2^, and 56 (43%) intensive (net-covered houses) type, which occupies an area of 93,000 m^2^. Generally, snail farming systems can be open field farms or covered constructions of different types [1,2,10,12,13,14]. The open-field system is extensive farming, where outdoor breeding and fattening take place. A net separates the divided sections in the field and protects snails from predators. Each section is planted suitably for snail feeding. The covered constructions (e.g., net-covered houses) are an intensive farming system. A net-covered greenhouse is equipped with a pressurized water spray cooling system with an additional compound diet provided. The duration of the production cycle (reproduction, fattening, and harvest) in snail farming depends on the biological cycle of the cultured species and the farm area’s climatic conditions [1,2,3,10,13,14,15,16].

The majority of experimental work on snail production (growth and food consumption) have been conducted in laboratory conditions [16,17,18], with limited research conducted in commercial snail farms. For instance, the authors in Reference [14] noticed that snails exhibited higher food consumption and growth rate at low experimental densities with most individuals reaching the adult stage. Observations of the fattening phase in external parks in mixed rearing farms (reproduction under controlled conditions and growth in external parks) [19] indicated that a biotic load of 50 g/m^2^ of three weeks to one-month-old individuals (*Helix aspersa*) was optimum for growth. In five months, a production of about 3 kg per m^2^ with about 89% of snails reaching marketable size was obtained. Furthermore, Gonzalez et al. [20] analyzed the spatial allocation of adult *H. aspersa* at three densities in an outdoor system during the fattening stage.

Climatic fluctuations heavily influence terrestrial snails in the Mediterranean in aridity and temperature. Long periods of drought combined with high temperatures prevalent during the summer months constitute a major factor of stress for land snails. These climatic parameters can shape their activity cycles and metabolic responses [21,22]. Environmental variables and other natural factors are emphatically seasonal in Greece and, hence, gastropods display anticipated oscillations in their activity. In addition, geographically distant snail populations can adopt distinctive life-history patterns due to adapting to the local climatic conditions [23,24]. Furthermore, environmental factors that control snails’ hibernation and arousals like temperature, humidity, and photoperiod differ due to geographical and climatic variation [22,25,26,27], and can influence snail productivity.

Many studies that have dealt with classification and typology in widespread animal farming systems have contributed to identifying the prevailing conditions of farms, leading to their significant improvement. For example, in Greece, small ruminants dairy farming, which constitutes 19.2% of total livestock production, has been amplified, leading to better animal welfare and hygiene conditions inside farms and greater milk yields [28]. These systems, of which significance is expected to rise in the future, are characterized by considerable capital investment, and their profitability is dependent on high ewe productivity [29]. Similarly, a study of pork production systems in Greece [30] exhibited their evolution from a family-type enterprise to an industrialized internal type.

The ever-increasing environmental impact of livestock production has called for fundamental changes in the demand of meat products and alternative production systems. Farmed snails could potentially constitute an environmental alternative to usual macro-livestock [2]. A comparison of studies of the most common meat production systems (cattle, pig, and poultry) with snail farming indicated an essential reduction of impact in almost all heliciculture categories [30]. However, no surveys have been conducted on the production practices of heliciculture. This study’s goal was a thorough analysis and a detailed characterization of commercial snail farms in Greece, considering the unstructured development of the snail-farming sector over recent years [10,11]. The classification of the farms is important to identify aspects of production that require improvement. Additionally, snail farms’ characterization in Greece could help Southern European countries improve heliciculture [2,30,31,32]. We aimed to effectively describe snail farming systems in Greece to produce a significant insight into the sector’s current status. In addition, we assessed the effects and impacts of various parameters (farming system, region, equipment, and facilities) in annual productivity using a questionnaire developed to assess the farms’ structural and administration characteristics. This allowed the identification of specific farm components that require alteration and/or improvement in order to achieve profitable management. This study is the first step for establishing strategic planning policies for the sustainable development of snail farming.

## 2. Materials and Methods

### 2.1. Area of Experiment

**Figure 1:**
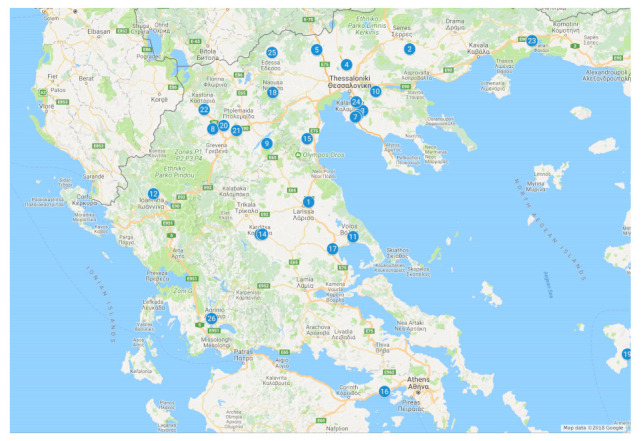
Map of snail farm location used in this survey.

The present research was conducted on 29 snail farms located in six regions (Central Macedonia, West Macedonia, Thessaly, Thrace, Western Greece, and Attica-Islands) in Greece (Figure 1). The climate in these regions is of the Mediterranean type with continental influence in some areas [33]. A widespread report of snail-farm systems and their attributes were collected through a designated questionnaire, holding visitations, and snail sampling.

### 2.2. Data Collection

Data were gathered with the use of a structured questionnaire through face-to-face interviews with snail producers. The duration of each interview was 2–3 h and each interviewee completed open-ended and closed questions. Data on management practices, technical characteristics, financial data, and production chain were collected. For the compilation of the questionnaire, related surveys [9,10,28,34] were considered, adapted to the current study’s requirements.

The data collected included seven main topics, further divided into sub-categories, the most important of which are: (a) Owner (Farmer’s experience, Farmer’s age), (b) Livestock (farmed snail species, snail weight, number of snails/kg, growth rate, mortality rate), (c) Production (farm production, productivity per beneficial surface), (d) Land (altitude, farming area, beneficial surface, substrate, plants), (e) Management and Nutrition (duration of operation, duration of reproduction period, rearing duration, starting period, harvest period, feed quantity, feeding frequency, use of cooling system, and shade rate of net), and (f) estimated income. Finally, questions (g) about farming type, equipment, and facilities were included in the survey.

Additional information was collected on each snail farm’s type of substrate and the use of growth rate indicators. A snail farm substrate included soil with planted broadleaf plants and gravel or soil without a plant presence. Substrate and diet were clustered on two categories: (a) type of substrate and (b) type of diet. More specifically, the substrate cluster included sub-clusters for (G-gravel, P-soil with cultivated plants, and S-soil). The later cluster included sub-clusters for diet types: (c–compound diet, cp-compound diet and plants, and p-plants). Growth rate indicators were used to detect possible effects of certain factors with daily fluctuation, such as climatic parameters (temperature, humidity, rainfall) on snail size. The presence of certain facilities (harvest warehouse, hatchery, packaging laboratory, and cooling chamber) and equipment (fabric for low temperatures, cages, tatters, egg storage containers, hibernation chamber, temperature sensors, humidity sensors, packaging materials, and agricultural tools) were evaluated either as existing or absent.

### 2.3. Sampling Procedures

The sampling period lasted from June to November 2017. In each farm, one sampling took place and 3 kg of commercial sized snails were collected in order to assess productivity components. Snails were numbered and weight was measured individually. The long sampling period is based on the necessity that snails in each farm must reach a marketable size. The produced snail species are *Cornu aspersum maximum* (syn. *Helix aspersa maxima*, common name “gros gris”) and *Cornu aspersum aspersum* (syn. *Helix aspersa*, common name “petit gris”). Each snail species’ marketable size was determined from the data received through the questionnaires (6 g for *Cornu aspesum aspersum* and 12 g for *Cornu aspersum maximum*).

### 2.4. Statistical Analysis

Initially, descriptive statistics (mean, median, standard deviation, and standard error) for continuous variables and frequencies for categorical variables were calculated. Thereafter, four original variables were selected (farming system, region, species, and substrate) and were used as factors to estimate their influence on three productivity variables (FP, Farm Production in kg × year ^−1^, PS, Productivity per surface in kg × (m^2^)^−1^ × year^−1^ and PB, Productivity per beneficial surface in kg × (m^2^)^−1^ × year^−1^). Descriptive statistics were calculated, and the main effect plots were presented. The small sample size justified the use of this statistical method.

For categorical variables, nonmetric Multidimensional Scaling (nMDS), based on the Bray–Curtis similarity index using the Unweighted Pair Group Method with Arithmetic mean (UPGMA) [35], was used as a means of visualizing the percentage of similarity between different farms. The percentage of similarity between farms was analyzed using cluster analysis based on the Bray–Curtis similarity index [36]. To normalize data and avoid skewness, a fourth root transformation was applied to the data prior to calculating similarities [37]. Data analysis was performed with the PRIMER package [38] (PRIMER-e, Auckland, New Zealand).

In order to estimate the potential income of snail farmers, total farm production (kg × year^−1^) was calculated with the price of 5 € per kg of fresh live snails, which is the current market price in Greece according to information collected through the questionnaires, which is in agreement with recent literature [10].

## 3. Results

### 3.1. Snail Farm Attributes

The average values for the structural and management characteristics for the total sampled snail farms are presented in Table 1. The snail farms’ average land was 3838 m^2^, comprised of 2593 m^2^ of the beneficial area and 1250 m^2^ of a non-beneficial area (Table 1). The farm installed at the highest altitude (759 m) above sea level was in Western Macedonia, while in Western Greece, a farm is located at sea level. The average farm operation duration exceeded eight months and the mean annual production was 1597 kg of fresh live snails (Table 1).

Most farmers belong to 30–40 years old (45%) and have obtained a degree from a higher education institution (77%). Heliciculture is not their only income source, as an overwhelming percentage (87%) has a second occupation. The longest farmers’ experience was five years (Table 1).

### 3.2. Farming Systems

Snail farming systems can be held in open field farms or covered constructions of different types. Apart from the two systems above, the present study recorded three categories of farming systems. Thus, five clusters were created with the following characteristics (Table 2).

Table 2 indicates that Greece’s most widespread systems are the net-covered greenhouse (38%) and the open field (38%). On the contrary, the least common systems are the mixed system with an open field and the elevated sections (7%). Open-field system farms are mainly located in West Macedonia (45%). The majority of net-covered greenhouses are found in Central Macedonia (45%), while several operate in Thessaly (27%).

From the 29 farms surveyed, 25 use *Cornu aspersum maximum* as their farmed species, whereas two farms use *Cornu aspersum aspersum*. Two farms culture both species (Table 2). The annual production process includes reproduction of the mature snails, hatching of eggs, and increase of hatchlings as well as fattening of snails. After the fattening is completed, snails are harvested.

Mixed systems with a net-covered greenhouse had the smallest available farming areas (Table 3). As far as altitude is concerned, elevated sections and net-covered greenhouses are higher than the other three types (Table 3). Regarding productivity (snails/kg), three types of farms are similar (elevated sections, a mixed system with a net-covered greenhouse, and a mixed system with an open field). The growth rate ranged between 0.09 and 0.15 gr × day^−1^ × snail^−1^ (Table 3). In the section of management, the duration of operation decreases in open fields (Table 3). In farm types with a net, it reaches up to nine months. In addition, the duration of the reproduction period has the same pattern (Table 3). In most of the farms, March was the starting month, while harvest took place in November.

**Figure 2:**
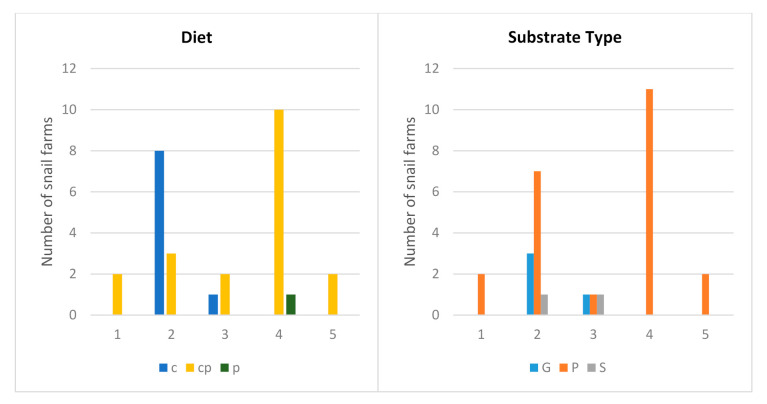
Frequencies for categorical variables (Substrate and Diet) in every farming system. Diet type: c—compound diet, p—plants, cp—compound diet and plants. Substrate type: G—gravel, P—soil with plants, S—soil. Farm type: 1—elevated sections, 2—net-covered greenhouse, 3—mixed system with a net-covered greenhouse, 4—open field, and 5—mixed system with an open field.

In total, 65% of snail farmers used a compound diet for snails, together with existing plants (Figure 2). The combination of a compound diet with plants was the most common diet in every farm type. Feeding only with plants appeared in one open farm (Figure 2). As a substrate for farmed snails, soil with cultivated broadleaf plants was mostly used (77%), along with gravel, exclusively in some net-covered greenhouses or soil without the presence of plants (Figure 2). Soil and gravel were only present in net-covered greenhouses or a mixed system with a net-covered greenhouse (Figure 2).

In Table 4, frequencies of facilities and equipment of different types are presented. Almost every snail farm has a harvest warehouse. Hatcheries were found only in mixed system farms (Table 4). Packaging stations and cooling chambers were not present in the minority of the farms (Table 4).

**Figure 3:**
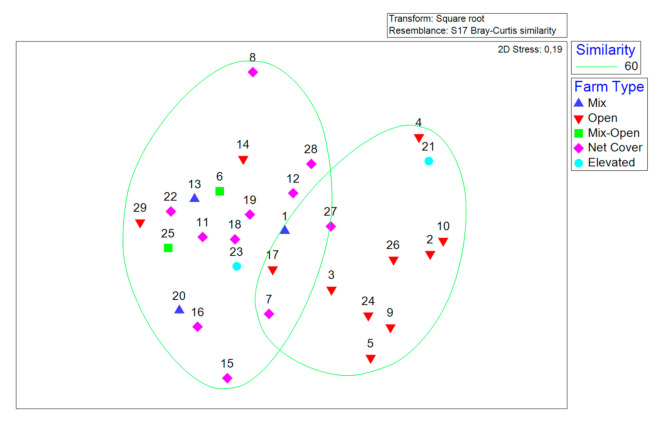
Multidimensional scaling ordination plot of the level of similarity between different farms (*n* = 29), based on the Bray–Curtis similarity index of square root-transformed indices (categorical variables). Closer points indicate higher similarity. Ellipses indicate groups with 60% similarity.

Figure 3 displays the extent of the similarity between different farming systems based on qualitative variables (presence or absence of equipment and facilities). The nonmetric multidimensional scaling (nMDS) plot indicated a high level of similarity (60% similarity based on the Bray–Curtis similarity index of square root-transformed indices) between most of the open field farms (Figure 3).

### 3.3. Production Variables

**Figure 4:**
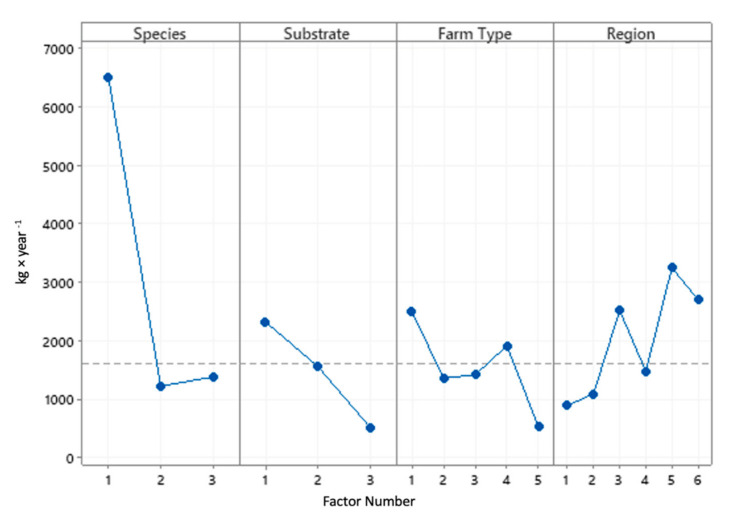
Main effects plot of each factor (species, substrate type, farm type, and region) exerts on the mean Farm Production (kg × year^−1^).

The descriptive statistics and the main effects plot of each factor’s influence (species, substrate type, farm type, and region) exerts on farm production (kg × year ^−1^) are presented in Table 5 and Figure 4, respectively.

**Figure 5:**
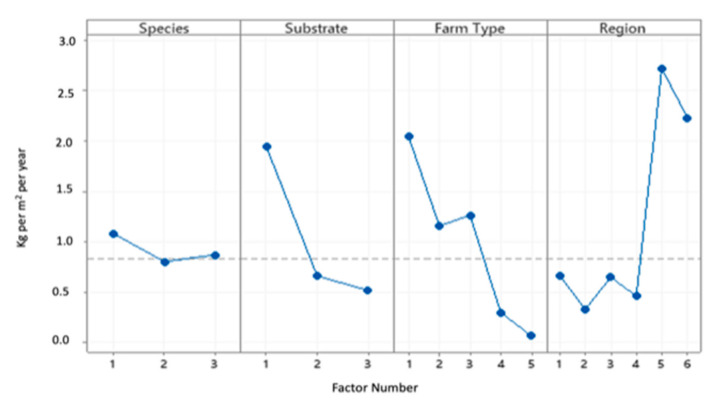
Main effects plot of the influence that each factor (species, substrate type, farm type, and region) exerts on the mean productivity per surface (kg × (m^2^)^−1^ × year^−1^).

Table 6 and Figure 5 indicate that the mean productivity per surface (kg × (m^2^)^−1^ × year^−1^) decreases from elevated sections to the mixed system with an open field. Regarding the region, data are grouped in three different clusters: regions 5 and 6 (Attica Islands and Western Greece) with high production, regions 1 and 3 (Thessaly and Central Macedonia) with medium production, and regions 2 and 4 (West Macedonia and Thrace) with low production (Table 6, Figure 5). It should be noted that all farms present in regions 5 and 6 are covered (three net-covered greenhouses and one with elevated sections). Furthermore, in Southern Greece (Attica), farms operate from February to November, whereas, in Northern Greece (Thrace), the operation starts in April or even in May. West Macedonia and Thrace farms operate for a shorter period throughout the year. In addition, an important fact is that seven out of eight farms located in these regions are the open field type.

**Figure 6:**
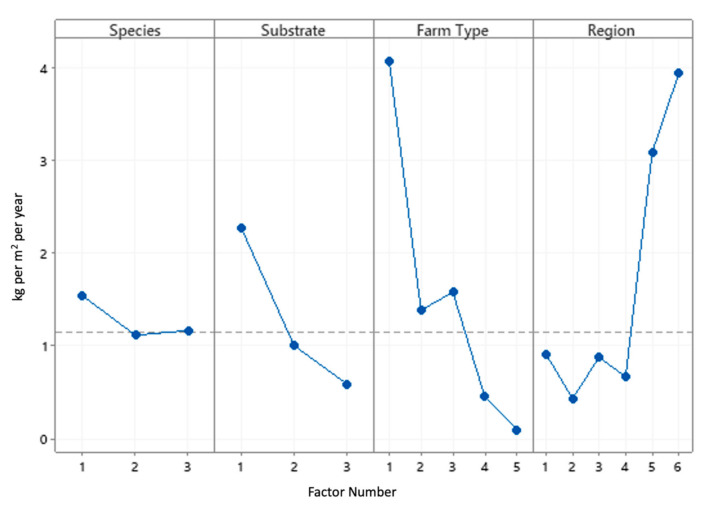
Main effects plot of the influence of each factor (species, substrate type, farm type, and region) on the mean productivity per beneficial surface (kg × (m^2^)^−1^ × year^−1^).

Mean production per beneficial surface (kg × (m^2^)^−1^ ) × year^−1^) in Table 7 follows the same pattern as the productivity per surface for all factors. The difference, however, is that each value is higher (Figure 6).

## 4. Discussion

The current study indicated that the average snail farmer’s age is 44.5 years with only five to six years of experience in this field. Heliciculture is not their only income source, as an overwhelming percentage (87%) has a second occupation. This result can be attributed to the fact that snail farming is a relatively new livestock industry sector [10,11]. The average land that snail farms occupy was 3838 m^2^, comprised of 2593 m^2^ beneficial areas and 1250 m^2^ non-beneficial areas. According to the latest available data from the Greek Ministry of Rural Development and Food [11], snail farms’ average size is small (8200 m^2^ for the open farms and 1800 m^2^ for the net-covered greenhouses), while very few have a size larger than 15,000 m^2^. The average farm operation duration exceeded eight months, and the mean annual production was 1597 kg of fresh live snails. Similar to other European countries [8,14,32], the main species produced in Greece are *Cornu aspersum aspersum* and *Cornu aspersum maximum,* which are both of a recognized commercial value [5,7,8,10,11,15].

This study classified 29 commercial snail farms in Greece and described them in detail (typology). The outcome indicated that heliciculture exhibits various classification schemes from extensive of small demand to intensive of high producing and investing farms. The present study recorded five farming systems, namely elevated sections (intensive), net-covered greenhouse (intensive), a mixed system with a net-covered greenhouse (intensive), an open field (extensive), and a mixed system with an open field (semi-intensive). Some have been described in previous studies [10,15]. Greece’s most widespread systems are the net-covered greenhouse (38%) and the open field (38%). Most of the net-covered greenhouses are found in Central Macedonia (45%), and are followed by Thessaly (27%). Open field farms are mainly located in West Macedonia (45%). A mixed system with an open field has the lowest production (528.5 kg × year^−1^) while the other systems’ production is similar. Annual farm production is directly related to the size of the fattening area. Although, the productivity per surface seems to decrease from elevated sections (2.04 kg × (m^2^)^−1^ × year^−1^) to the mixed system with an open field (0.067 kg × (m^2^)^−1^ × year^−1^). Finally, higher values were obtained for productivity per beneficial surface (maximum 4.077 kg × (m^2^)^−1^ × year^−1^ for elevated sections and minimum 0.101 kg × (m^2^)^−1^ × year^−1^ for a mixed system with an open field). This indicator is much more accurate about the actual area where snails can disperse. Results showed that intensive farms with higher facilities scores have higher productivity, in agreement with previous experiments, in which all animals produced under laboratory conditions, become adults. In contrast, when fattening occurred in greenhouses, adults were 80% of the total production [39]. Furthermore, the small sample size limits the evaluation of the management and structural components that affect farm production.

The duration of the annual operation lasts between seven and nine months. According to other studies, under intensive rearing, marketable size takes four to five months [17]. Surely, the period of rearing could be expanded, which is not necessarily ideal. This increase (more than five months) has been proven to slow down snail growth and decelerate their adulthood [14]. The productivity per beneficial surface (0.58–6.15 kg × (m^2^)^−1^ × year^−1^) was smaller than the one reported by Reference [13] (1.19–2.75 kg × (m^2^)^−1^ × year^−1^) in mixed farming (reproduction in a controlled building and fattening in an outdoor park). Farmers use soil with growing plants, mostly as a substrate. As described in previous studies [1,40], snails fed only with green vegetables had slower growth and, at the end of the experiment, weighed eleven times lower than the ones fed with the compound ailment.

Snail farms in our research were dispersed in six different regions (Thrace, Central Macedonia, West Macedonia, Thessaly, Western Greece, and Attica-Islands) from low to high altitudes (759 m) and operated under highly variable temperature regimes. In those different localities and regions of Greece, there is a wide assortment of climatic types, portrayed by critical contrasts in the span and power of wet and dry periods [41]. The farms in Attica—Islands and Western Greece have the highest production because of the ideal climatic parameters. It should also be noted that all the farms in these regions were covered (three net-covered greenhouses and one elevated section). Because of low temperatures, West Macedonia and Thrace farms operate for a shorter period throughout the year. Seven out of eight farms in these regions are of the open field type, making them more vulnerable to local climatic conditions. Previous studies have shown that geographically distant natural snail populations can adopt distinctive life-history patterns due to adaptation to the local environmental conditions [23,24].

Compared to other agricultural systems that have long been established, snail farming in Greece is still evolving, and the present classification can aid farmers in deciding which method is more efficient both geographically and in terms of productivity. On the other hand, pork production systems in Greece have already evolved, from a family-type enterprise (herd size of 10–20 shows) to an industrialized, internal type with a remarkably large livestock number [30]. The same evolution was displayed in sheep, whereas, in the last decade and due to changing socioeconomic conditions, traditional systems have been replaced by others, characterized by a considerable capital investment and high ewe productivity [29]. In addition, dairy farmers changed their small-scale farms to an entrepreneurial livestock breeding activity. This action has facilitated the substantial improvement of the conditions under which dairy farms operate [28].

## 5. Conclusions

The assessment of modern farms’ structural and management characteristics, including heliciculture, can contribute to sustainable food production. This is the first study that classifies snail farms in Greece and describes them in detail. Five farming systems (elevated sections, net-covered greenhouse, mixed system with net-covered greenhouse, open field, and mixed system with the open field) were identified. Results indicated that intensive farms exhibit high production. Geographical location affects production and influences the duration of operation, especially in open field farms. Snail farms in Northern Greece are forced to operate for a shorter period throughout the year.

Snail farming can be a potentially promising business, but this depends on a multitude of factors. We need more in-depth scientific knowledge and research on the breeding and growth of snails and the climatic and geographical aspects of the selected areas of farm settlement. The critical factors identified and the promotion of the product in national and international markets will guarantee business sustainability.

## Figures and Tables

**Table 1:** Structural and administration features of the 29 snail farms (mean, standard error, standard deviation, minimum, median, and maximum).

Variable	Number	Mean	SE	St. Dev.	Min	Median	Max
Farmer’s Experience (years)	29	4.83	0.43	2.32	1	5.00	13
Farmer’s age (years)	29	44.14	1.63	8.77	31	45	60
Growth rate (gr × day^−1^ × snail^−1^)	29	0.11	0.007	0.04	0.059	0.096	0.1930
Number of Snails × kg^−1^	29	79.51	3.98	21.43	54.41	75.30	168.07
Number of snails (year)	29	138,043	40,046	215,654	8636	75,851	1,120,381
Snail weight (gr)	29	13.13	0.48	2.58	5.95	13.28	18.38
Mortality (%)	19	22.1	19.2	10.4	3	25	50
Altitude (m)	29	223.8	46.4	249.7	0	108	759
Farming area (m^2^)	29	3838	839	4518	500	1500	16,000
Beneficial surface (m^2^)	29	2593	530	2853	350	1200	10,500
Duration of operation (months)	29	8	0.291	1.566	4	8	10
Duration of reproductive period (days)	29	158.28	5.75	30.95	120	150	210
Rearing Duration (days)	29	241.52	8.69	46.78	120	240	270
Dry Feed Quantity (kg × year^−1^)	25	1628.86	404.15	2194.84	100	915	10,000
Cooling system (min × day^−1^)	29	22.07	1.7	9.15	6	20	45
Shade rate of net (%)	17	72.94	2.539	10.467	50	70	90
Farm Production (kg × year−^1^)	29	1597	375	2018	100	1000	10,005
Productivity (kg × m^2^)^−1^ × year^−1^)	29	0.829	0.185	0.996	0.035	0.400	4.435
Prod. per Ben. Surf. (kg × (m^2^)^−1^ × year^−1^)	29	1.148	0.264	1.420	0.058	0.583	6.154
Estimated income (€ × year−^1^)	29	7281	1839	9903	500	3750	50,000

**Table 2:** Classification of snail farming systems recorded in the present study.

Farm System	Ν (%)	Main Facilities	Rearing Stages	Species
Elevated sections	2 (7%)	Elongated raised panel boxes separated for reproduction and fattening	Reproduction, hatching and fattening of a brood, under semi—natural environment	*Cornu aspersum maximum*
Net—covered greenhouse	11 (38%)	Net-covered greenhouse. Inside, divided sections separated by net	Reproduction and fattening under semi—natural environment	*Cornu aspersum maximum* *(9 farms),* *Cornu aspersum aspersum* *(1 farm),* *Both (1 farm)*
Mixed system with net-covered greenhouse	3 (10%)	Hatchery Net-covered greenhouse	Reproduction and hatching under a controlled environment, fattening under a semi-natural environment	*Cornu aspersum maximum*
Open field	11 (38%)	Open field. Divided sections separated by net	Outdoor reproduction and fattening	*Cornu aspersum maximum* *(9 farms)* *Cornu aspersum aspersum* *(1 farm)* *Both (1 farm)*
Mixed system with open field	2 (7%)	Hatchery Open field	Reproduction and hatching under a controlled environment. Outdoor Fattening	*Cornu aspersum maximum*

**Table 3:** Mean value ± standard deviations (S.D.) for continuous variables in every farming system.

		1 (*n* = 2)	2 (*n* = 11)	3 (*n* = 3)	4 (*n* = 11)	5 (*n* = 2)
	Attributes	Mean	Mean ± SD	Mean ± SD	Mean ± SD	Mean
Staff	Experience of farmer (years)	4	5.45 ± 3.05	3.3 ± 2.1	4.8 ± 1.8	4.5
	Farmer’s age (years)	43.5	40.1 ± 9.5	44.3 ± 1.15	46.3 ± 8.5	55
Livestock	Growth rate (gr × day^−1^ × snail^−1^)	0.15	0.1 ± 0.03	0.14 ± 0.04	0.09 ± 0.04	0.14
	Number of snails/kg	75.3	79.9 ± 30.4	68.1 ± 4.89	84.9 ± 13.9	68.8
Land	Altitude (m)	554.5	305.36 ± 279	64 ± 54.4	138 ± 195.2	156
	Farming area (m^2^)	3150	4200 ± 4526.6	1583.3 ± 1233.2	4414.5 ± 5682.4	5000
	Beneficial surface (m^2^)	2270	3074.5 ± 3262.3	1166.7 ± 814.5	2725.6 ± 3208.6	3250
Management	Duration of operation (months)	9	8.36 ± 1.69	8	7.9 ± 1.81	7
	Duration of reproduction period (days)	180	171.8 ± 27.1	160 ± 45.8	144.5 ± 29.4	135
	Rearing duration (days)	270	254.5 ± 48.8	240	240 ± 55.3	210
	Dry feed quantity (kg × year^−1^)	850	2080 ± 2879.9	609.3 ± 447.9	1607 ± 1997.7	760
	Farm production (kg × year^−1^)	2500	1358.7 ± 1611.3	1416.7 ± 520.4	1913.6 ± 2779.4	528.5
	Estimated income (€ × year^−1^)	3500	3849.5 ± 1857.9	6996.7 ± 9570	11,400 ± 14,397	5363.7

Farm type: 1—elevated sections. 2—net-covered greenhouse. 3—mixed system with a net-covered greenhouse. 4—open field. 5—mixed system with open field.

**Table 4:** Frequencies for categorical variables (facilities and equipment) in every farming system.

	1 (*n* = 2)	2 (*n* = 11)	3 (*n* = 3)	4 (*n* = 11)	5 (*n* = 2)
Facilities					
Harvest Warehouse	2	10	3	10	2
Hatchery	0	0	2	0	1
Packaging station	1	2	1	2	0
Cooling chamber	1	3	1	2	2
Equipment					
Fabric for low temperature	2	6	1	9	0
Shelters (wooden)	1	10	3	6	2
Cages	0	3	1	2	1
Tatters	1	6	2	6	1
Egg storage containers	0	0	2	0	1
Temperature and humidity sensors	0	7	1	2	2
Packaging materials	1	7	2	6	2
Agricultural tools	1	9	3	10	2

Farm type: 1—elevated sections, 2—net-covered greenhouse, 3—mixed system with a net-covered greenhouse, 4—open field, 5—mixed system with an open field.

**Table 5:** Descriptive statistics of farm production (kg × year ^−1^) for all factors (species, substrate type, farm type, and region).

Factor	Number	Mean	SE	St. Dev.	Min	Median	Max
Species	(n)						
1	2	6503			3000	6503	10,005
2	25	1221	251	1254	100	735	5500
3	2	1382			1360	1382	1404
Substrate	(n)						
1	4	2326	1108	2217	400	1702	5500
2	23	1563	432	2073	100	1000	10,005
3	2	521			170	521	872
Farm type	(n)						
1	2	2500			1000	2500	4000
2	11	1359	486	1611	100	872	5500
3	3	1417	300	520	1000	1250	2000
4	11	1914	838	2779	400	735	10,005
5	2	528.5			500.0	528.5	557.0
Region	(n)						
1	11	890	170	563	100	700	2000
2	6	1092	289	709	400	907	2000
3	6	2518	1556	3812	170	868	10,005
4	2	1475			450	1475	2500
5	2	3250			1000	3250	5500
6	2	2702			1404	2702	4000

Snail species: 1—Cornu aspersum aspersum, 2—Cornu aspersum maximum, 3—C. a. aspersum and C. a. maximum. Substrate: 1—gravel, 2—soil with plants, 3—soil. Farm type: 1—elevated sections, 2—net-covered greenhouse, 3—mixed system with a net-covered greenhouse, 4—open field, 5—mixed system with open field. Region: 1—Central Macedonia, 2—West Macedonia, 3—Thessaly, 4—Thrace, 5—Western Greece, 6—Attica Islands.

**Table 6:** Descriptive statistics of Productivity per surface (kg × (m^2^)^−1^ × year^−1^) for all factors (species, substrate type, farm type, and region).

Factor	Number	Mean	SE	St. Dev.	Min	Median	Max
Species	(n)						
1	2	1.083			0.667	1.083	1.500
2	25	0.805	0.211	1.056	0.035	0.367	4.435
3	2	0.872			0.368	0.872	1.376
Substrate	(n)						
1	4	1.938	0.870	1.740	0.400	1.457	4.435
2	23	0.663	0.158	0.759	0.035	0.367	3.077
3	2	0.521			0.170	0.521	0.872
Farm type	(n)						
1	2	2.04			1.00	2.04	3.08
2	11	1.159	0.379	1.258	0.170	0.872	4.435
3	3	1.263	0.156	0.269	1.000	1.250	1.538
4	11	0.2981	0.0777	0.2576	0.0564	0.2333	0.8333
5	2	0.0674			0.0348	0.0674	0.1000
Region	(n)						
1	11	0.668	0.200	0.663	0.035	0.400	2.133
2	6	0.328	0.190	0.466	0.056	0.108	1.250
3	6	0.651	0.213	0.521	0.170	0.517	1.500
4	2	0.462			0.090	0.462	0.833
5	2	2.72			1.00	2.72	4.44
6	2	2.227			1.376	2.227	3.077

Snail species: 1—*Cornu aspersum aspersum*, 2—*Cornu aspersum maximum*, 3—*C. a. aspersum* and *C. a. maximum.* Substrate: 1—gravel, 2—soil with plants, 3—soil. Farm type: 1—elevated sections, 2—net-covered greenhouse, 3—mixed system with a net-covered greenhouse, 4—open field, 5—mixed system with open field. Region: 1—Central Macedonia, 2—West Macedonia, 3—Thessaly, 4—Thrace, 5—Western Greece, and 6—Attica- Islands.

**Table 7:** Descriptive statistics of productivity per beneficial surface (kg × (m^2^)^−1^ × year^−1^) for all factors (species, substrate type farm type, and region).

Factor	Number	Mean	SE	St. Dev.	Min	Median	Max
Species	(n)						
1	2	1.544			1.213	1.544	1.875
2	25	1.115	0.304	1.518	0.058	0.479	6.154
3	2	1.167			0.613	1.166	1.721
Substrate	(n)						
1	4	2.268	0.941	1.881	0.5	3.402	4.928
2	23	1.002	0.279	1.338	0.058	0.525	6.154
3	2	0.591			0.213	0.146	0.969
Farm type	(n)						
1	2	4.077			2	1.029	6.154
2	11	1.386	0.427	1.416	0.213	0.5	4.928
3	3	1.579	0.194	0.337	1.250	1.25	1.923
4	11	0.452	0.124	0.411	0.071	1.212	1.213
5	2	0.101			0.058	0.142	0.143
Region	(n)						
1	11	0.914	0.265	0.879	0.058	0.525	2.667
2	6	0.434	0.235	0.577	0.071	0.521	1.563
3	6	0.877	0.274	0.672	0.213	0.251	1.875
4	2	0.67			0.150	1.642	1.190
5	2	3.089			1.250	2.949	4.928
6	2	3.938			1.721	3.106	6.154

Snail species: 1—*Cornu aspersum aspersum,* 2—*Cornu aspersum maximum*, 3—*C. a. aspersum* and *C. a. maximum.* Substrate: 1—gravel, 2—soil with plants, 3—soil. Farm type: 1—elevated sections, 2—net-covered greenhouse, 3—mixed system with a net-covered greenhouse, 4—open field, 5—mixed system with an open field. Region: 1—Central Macedonia, 2—West Macedonia, 3—Thessaly, 4—Thrace, 5—Western Greece, 6—Attica-Islands.

## Data Availability

Not applicable.
